# Static mechanical analysis of the vertebral body after modified anterior cervical discectomy and fusion (partial vertebral osteotomy): a finite element model

**DOI:** 10.1186/s13018-023-04033-8

**Published:** 2023-08-01

**Authors:** Huo-huo Xue, Dian Tang, Wen-han Zhao, Liang Chen, Zhong Liao, Jing-lai Xue

**Affiliations:** 1 Department of Spine Surgery, Fuzhou Second Hospital, 350007 Fuzhou, China; 2 Fujian Medical University Union Hospital, Fuzhou, 350100 China

**Keywords:** Modified anterior cervical discectomy and fusion (Mod ACDF), Posterior longitudinal ligament ossification, Finite element analysis

## Abstract

**Background:**

Modified anterior cervical discectomy and fusion (Mod ACDF) can effectively address ossification of the posterior longitudinal ligament (OPLL), which is difficult to remove directly from the posterior edge of the vertebral body, with considerably lesser damage as compared to anterior cervical corpectomy and fusion (ACCF). We compared the static mechanics of different anterior approaches by using an ideal finite element model.

**Methods:**

A complete finite element model was established and classified into the following three surgical models according to different model cutting operations: ACDF, ACCF, and Mod ACDF. Three different bone volume situations (normal bone mineral density, osteopenia, and osteoporosis) were simulated. After fixing the lower surface of C5 or C6, a load was applied to the upper surface of C4, and the stress distribution and displacement of the upper surface of C5 or C6 were observed and the related values were recorded.

**Results:**

The average Von Mises Stress and displacement levels of Mod ACDF were between those of ACDF and ACCF; with the peak Von Mises Stress occurring on the posterior side of the vertebral body (Points 1–4). The change in Von Mises Stress of the vertebral body is not significant during bone loss. However, the degree of displacement of the vertebral body surface and risk of vertebral collapse are increased (100 N: 13.91 vs. 19.47 vs. 21.62 μm; 150 N: 19.60 vs. 29.30 vs. 31.64 μm; 200 N: 28.53 vs. 38.65 vs. 44.83 μm).

**Conclusions:**

The static biomechanical effects caused by Mod ACDF are intermediate between ACDF and ACCF, and the risk of vertebral body collapse is lower than that by ACCF. Therefore, Mod ACDF may be an effective solution when targeting OPLL with poorly positioned posterior vertebral body edges.

## Background

Ossification of the posterior longitudinal ligament (OPLL) is a rare soft-tissue ectopic ossification disease with a pathological process involving multiple genetic targets and signaling pathways [1–3]. Current studies suggest that surgical intervention is the key to treating OPLL. According to a guideline [4], direct surgical removal of single-segment osteoid blocks that do not exceed the K-Line is recommended for the ventral approach [anterior cervical discectomy and fusion (ACDF) and anterior cervical corpectomy and fusion (ACCF)], whereas, for cases of OPLL exceeding the K-Line, a posterior approach is more necessary (laminectomy and laminoplasty). The anterior cervical approach involves multilayered muscle and fascia stripping with important large vessels and tracheal accompaniment, and the surgical difficulty and complications are considerably higher than those of the dorsal approach [5–8].

Focal OPLL is usually found in areas with high ligament mobility, such as the posterior edge of the disk, an area more prone to physical strain, and inflammatory responses [9, 10]. According to Saito et al. [11], for ossified ligaments, the expression of interleukin-6 is either critical for ectopic ossification. Therefore, the case of ossification at the posterior edge of the central vertebral body with little distraction stimulation is extremely rare, and the surgical treatment criteria required by the guidelines do not apply. Previously, we reported a case of focal ossification at the posterior margin of the vertebral body [12], in which a modified ACDF (Mod ACDF) technique was used to partially osteotomize the diseased vertebra and remove it along with the ossified mass.

The patient's cervical spine indicators were still improving and gradually meeting the fusion criteria, which benefits from this patient's good bone mass. Before the start of this technique, we had only empirically determined that this man's vertebrae were strong enough to resist compression by gravity from above and would not collapse. However, there is no denying that the partial amputation of the vertebrae would alter the biomechanical structure of the head, which could have implications for patients potentially not eligible for this procedure. Considering these issues, we decided to perform a transverse static mechanical analysis using a finite element model to comprehensively evaluate the advantages and disadvantages of this new technique.

## Methods

### Build a complete finite element model of the cervical spine

A healthy 52-year-old man (height 168 cm, weight 70 kg) was recruited to exclude cervical spine-related diseases (e.g., fractures, deformities, tumors, spinal stenosis, and so on). His cervical spine was scanned continuously by computed tomography (CT) from the base of the skull to the seventh cervical vertebra, and 3D CT images of the cervical spine were extracted and saved as DICOM. The DICOM files were imported into Mimics Medical 21.0 (Materialise, Belgium) and modeled by the default orientation defined by the images. The image threshold was adjusted to separate and remove the vertebrae, other than C4–C6. The eraser function erased the abnormal connections of the adjacent vertebrae to ensure the independence of each vertebra. Erase editing was repeated to successfully build the cervical spine 3D model and export the STL file. Geomagic Wrap 2021 (USA) was opened and imported into the STL file, and after mesh redrawing and polishing, the 3D image of the cervical spine was curved and the result was saved in STP format. The cage shape was set as a hexahedral rectangular structure with a pointed tip on one side; the titanium mesh was a metallic cylindrical mesh structure. The complete cervical spine is grouped into different surgical models.

### Surgical model design

#### ACCF

**Fig. 1:**
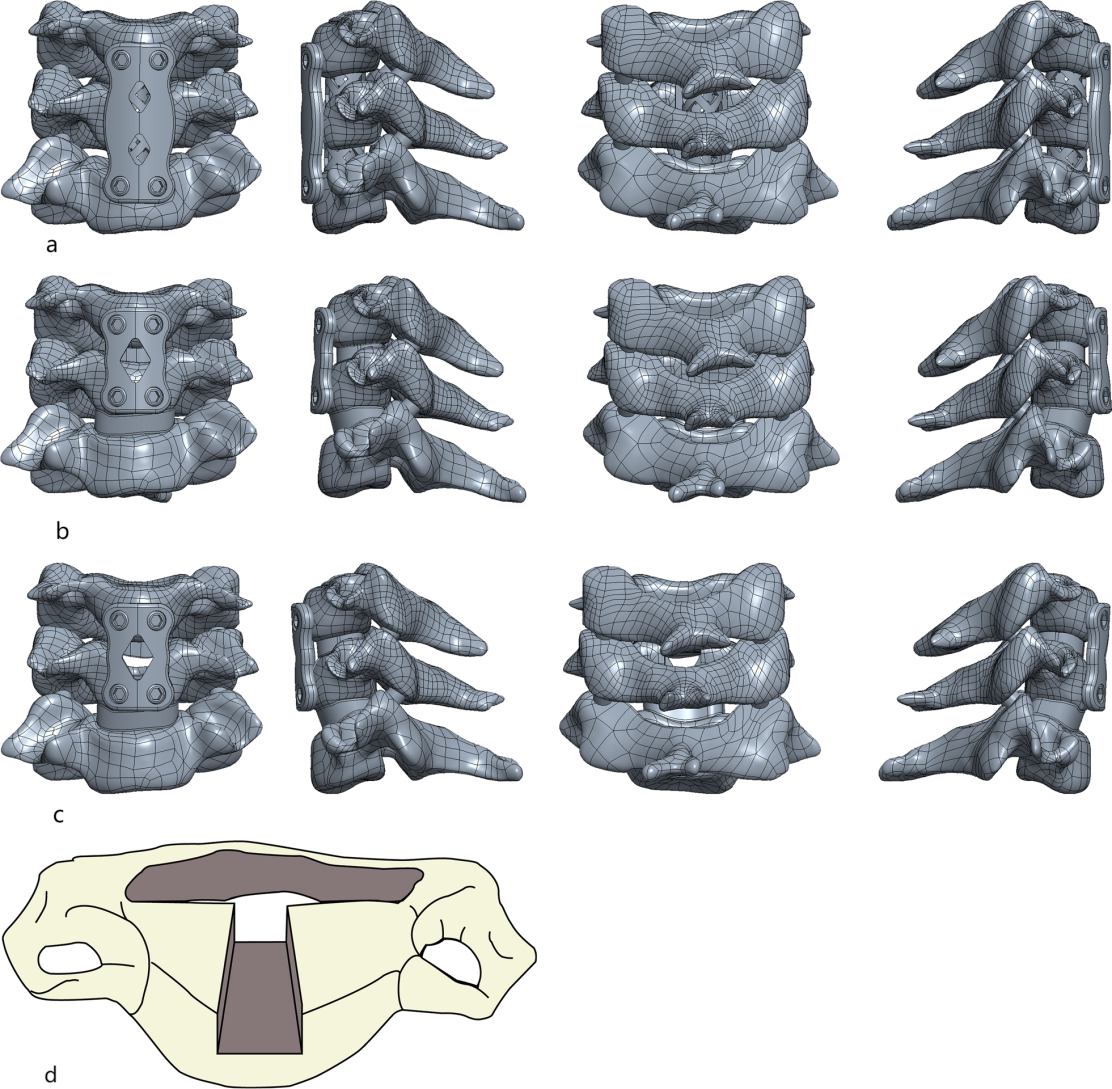
Finite element model of the cervical spine under three types of surgical techniques. **a** According to the ACCF surgical construction model, the width of the resected vertebral body was 15 mm, and a titanium mesh (10 × 50 mm, inner diameter 8 mm) was placed in the decompression zone, 1 mm from the anterior edge of the vertebral body. A titanium plate (1 × 22 × 16 mm) spanning three vertebral bodies was set on the anterior side of the C4–C6 vertebral body. **b** According to the ACDF surgical construction model, the cartilage endplate was scraped after removing the disk tissue, and a cage (5 × 14 × 16 mm) with a closely fitting bony endplate at both ends was placed. A plate (1 × 20 × 16 mm) spanning both vertebral bodies was set on the anterior side of the C4–C5 vertebral body and fixed with four screws at the head and tail ends. **c** Mimicking the ACDF technique, the cartilage endplate was scraped after the removal of the C4–5 intervertebral disk. A portion of the C5 vertebral body (8 × 9 mm) was excised, and a cage (5 × 14 × 16 mm) with closely fitting bony endplate at both ends was placed. A plate (1 × 20 × 16 mm) spanning both vertebral bodies was set anteriorly to the C4–C5 vertebral body and fixed with four screws at the cephalic and caudal ends and ensured that the screws were all buried in the cancellous bone; **d** the frontal view of the Mod ACDF osteotomy vertebral body

Mimicking the ACCF procedure, the C4/5 and C5/6 intervertebral disks were resected and the C4 inferior cartilage endplate, part of the C5 vertebral body, and the C6 superior cartilage endplate were removed from the top to the bottom. The width of the resected vertebral body was 15 mm. A LIBEIER titanium mesh (10 × 50 mm, inner diameter 8 mm) was placed in the decompression zone, 1 mm from the anterior edge of the vertebral body, and both sides of the titanium mesh were closely fitted to the bone. A titanium plate (1 × 22 × 16 mm) spanning three vertebral bodies was set on the anterior side of the C4–C6 vertebral body and fixed with four screws at the head and tail ends (Fig. 1a).

#### ACDF

Mimicking the ACDF procedure, the C4/5 disk was resected, the inferior cartilage endplate of C4 and superior cartilage endplate of C5 were removed, and the LIBEIER cervical cage (5 × 14 × 16 mm) was placed in the decompression zone, 1 mm from the anterior edge of the vertebral body, with both sides of the cage fitting closely to the bone. A plate (1 × 20 × 16 mm) was set across both vertebral bodies anteriorly to the C4–C5 vertebral body and fixed with four screws at the head and tail ends (Fig. 1b).

#### Mod ACDF

The adjacent cartilage endplate of the intervertebral space was scraped after the C4/5 disk resection following the previously reported technique, simulating the ACDF technique. A portion of the C5 vertebral body (8 × 9 mm) was excised, and a LIBEIER cervical cage (5 × 14 × 16 mm) was placed 1 mm from the anterior edge of the vertebral body, with both sides of the cage fitting closely to the bone. A plate (1 × 20 × 16 mm) was set across both vertebral bodies anteriorly to the C4–C5 vertebral body, and was fixed with four screws at the head and tail ends to ensure that the screws are all buried in the cancellous bone (Fig. 1c, d).

### Finite element analysis

Bone mass was classified as follows according to the diagnostic criteria for osteoporosis provided by WHO: *T* value ≥ − 1.0 SD for normal bone mineral density, − 2.5 SD < *T* value < − 1.0 SD for reduced bone mass, and *T* value ≤ − 2.5 SD for osteoporosis. Three additional subgroups were created under the surgical model, which were as follows: normal bone mass (NBM) group, osteopenia (OPA) group, and osteoporosis (OPS) group. Referring to Polikeit et al. [13] study, the elastic modulus of the cortical bone, end plate, and cancellous bone in the OPA group was reduced by 33% as compared to that of the OPS group, whereas the elastic modulus of the cortical bone and end plate in the OPS group was reduced by 33% and that of the cancellous bone by 66%.

**Fig. 2:**
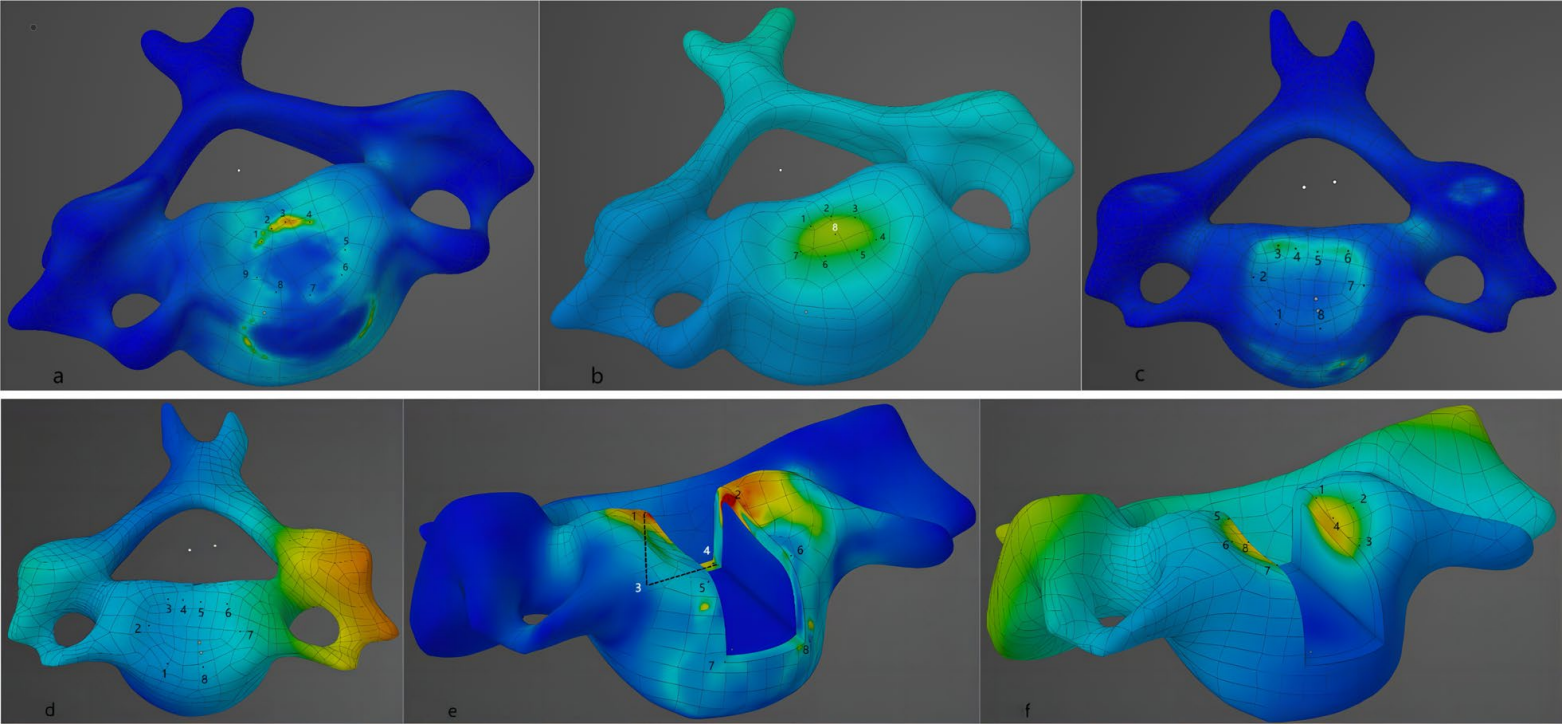
**a** The stress distribution on the upper surface of C6 under 200 N load in the normal bone-volume ACCF model group, with the maximum stress value occurring at point 3. The stress distribution on the posterior side of the vertebral body is significantly larger than that on the anterior side of the vertebral body due to partial load sharing by the anterior titanium plate. **b** Deformation state on the upper surface of C6 under 200 N load in the normal bone-volume ACCF model group, with the maximum deformation variable occurring at point 8, which is in the middle and posterior sides of the vertebral body. **c** The stress distribution on the upper surface of C5 in the normal bone-volume ACDF model group under 200 N load, with more concentrated stress on the left posterior side, and the maximum value appeared at point 3. **d** The deformation state on the upper surface of C5 in the normal bone-volume ACDF model group under 200 N load, with a more uniform overall deformation distribution. **e** The stress distribution on the upper surface of C5 in the normal bone-volume Mod ACDF model group under 200 N load, with the maximum value appearing at point 2, and the stress distribution at point 3. The maximum value appears at point 2, and the overall distribution of stress is on the posterior vertex and transverse edge of the notch; f. The deformation state of the upper surface of C5 under 200 N load in the normal bone weight Mod ACDF model group, the maximum value appears at points 8 and 4, and the possibility of a collapse of the vertebral body is greater in these two places

The assembled cervical spine model was assigned using ANSYS Workbench 2023 R1 (ANSYS, USA), material parameters were specified (Table 1), contact points were set, and cervical spine axial compression was simulated and calculated under static conditions. Based on the observation of the force performance of the titanium cage and cage after placement in the decompression slot, the stress concentration, and deformation areas under the three techniques were not consistent; the distribution is shown in Fig. 2. We assigned 16 points of maximum variation to each model group and recorded and compared the Von Mises Stress and deformation at each point separately.

**Table 1:** Spinal structure and instrumentation material properties

Spinal structure and instrumentation	Young’s modulus (MPa)	Poisson’s ratio
Cortical bone with normal bone mass	12,000	0.30
Cortical bone with osteopenia	8040	0.30
Cortical bone with osteoporotic	8040	0.30
Cancellous bone with normal bone mass	450	0.25
Cancellous bone with osteopenia	302	0.25
Cancellous bone with osteoporosis	149	0.25
Bony endplate	Same cortical bone value as normal/osteopenia/osteoporosis	
Cartilaginous endplate	25	0.4
Annulus	4.2	0.45
Nucleus pulposus	1	0.50
Intervertebral articular cartilage	35	40.00
Titanium mesh/titanium plate/screw	110,000	0.36
PEEK cage	3600	0.30

### Validity check

The validity of the present model was verified by comparing it with the results of previous experiments. For ACCF, the lower surface of the C6 vertebra was completely fixed with each node fixed, whereas C4 was not constrained and subjected to the load vector. For ACDF vs. Mod ACDF, the lower surface of the C5 vertebra was completely fixed with each node fixed, whereas C4 was not constrained and subjected to the load vector. By applying different preload sizes to the upper surface of C4 in the ACCF and ACDF groups, the results following the reports of Ouyang and Guo [14, 15] can be used for the experiments.

### Set the boundary conditions and apply the load method

The intervertebral fusion was binded to the vertebral body. For the binding boundary, C5 (ACDF, Mod ACDF) or C6 (ACCF) vertebral body was fully fixed in all directions at all nodes on the lower surface, C4/(C5) was not bound and received the load from the upper surface of C4. The assumed conditions were as follows. The material properties relevant to this study are assumed to be continuous and homogeneous. There is a frictionless surface contact between the joints, between the disk and vertebral body, and between the disk, and endplate. A preload of 100, 150, and 200 N was applied to the upper surface of C4 in each model group, and finite element simulations were performed to calculate the stress distribution and degree of vertebral body deformation under axial pressure after setting other conditions and to derive the risk of implant collapse between different techniques.

### Statistical analysis

Data were statistically analyzed using SPSS statistical software package (version 23.0; IBM Corporation, USA). An analysis of variance was used for inter-group and intra-group comparisons of normally distributed measures. Non-normally distributed measures were tested using nonparametric tests, and Kruskal–Wallis multiple samples were used for comparison between and within groups. *P* < 0.05 was considered a significant difference.

## Results

**Fig. 3:**
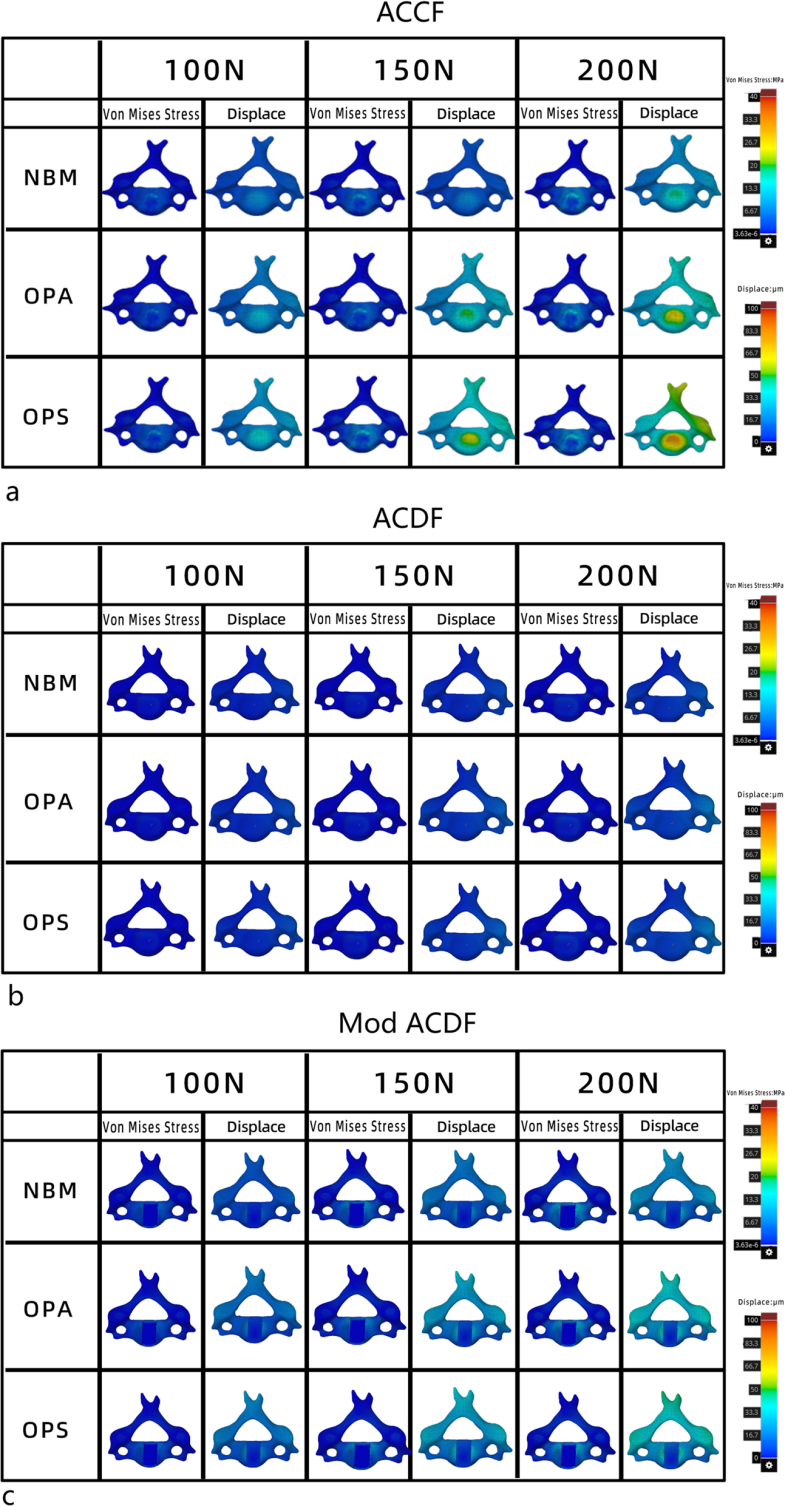
**a** FEA results of the ACCF model group; **b** FEA results of the ACDF model group; **c** FEA results of the Mod ACDF model group

**Fig. 4:**
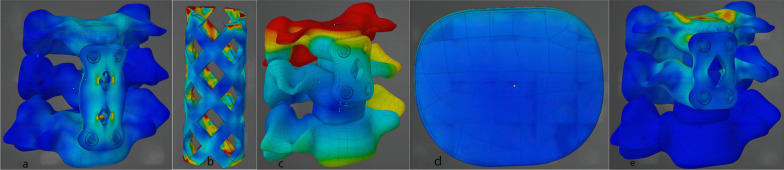
**a**, **b** Overall stress distribution of the ACCF model group: the stress on the anterior side is concentrated on the titanium plate, and the stress on the posterior side is concentrated on the mid-posterior side of the titanium mesh. **c** Overall stress distribution of the ACDF model group. **d** Elevation view of a cage in the ACDF model group **e** The stress of the Mod ACDF model group is concentrated on the posterior side of the notch and titanium plate

**Fig. 5:**
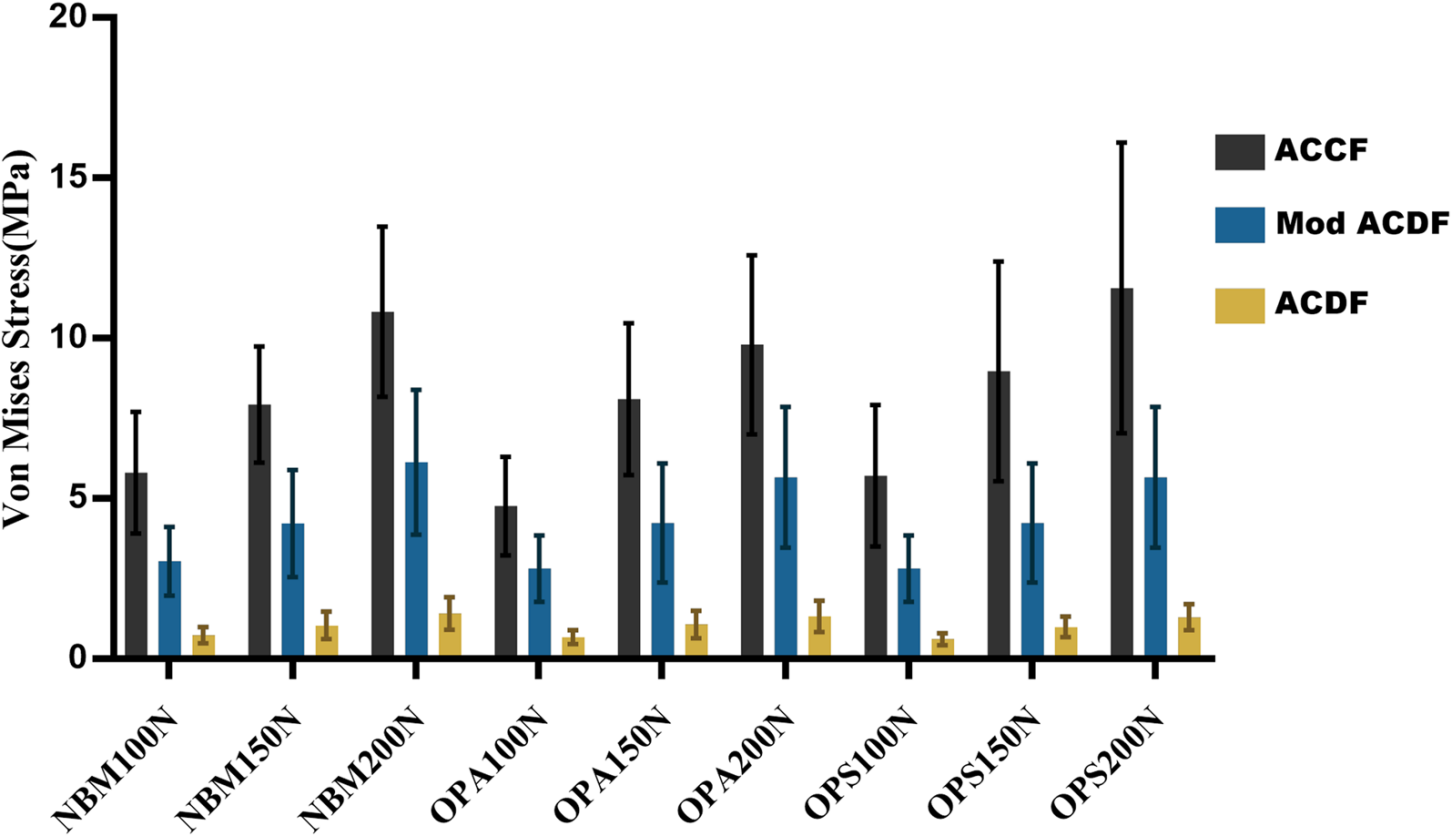
Comparison of Von Mises stress under different conditions

**Fig. 6:**
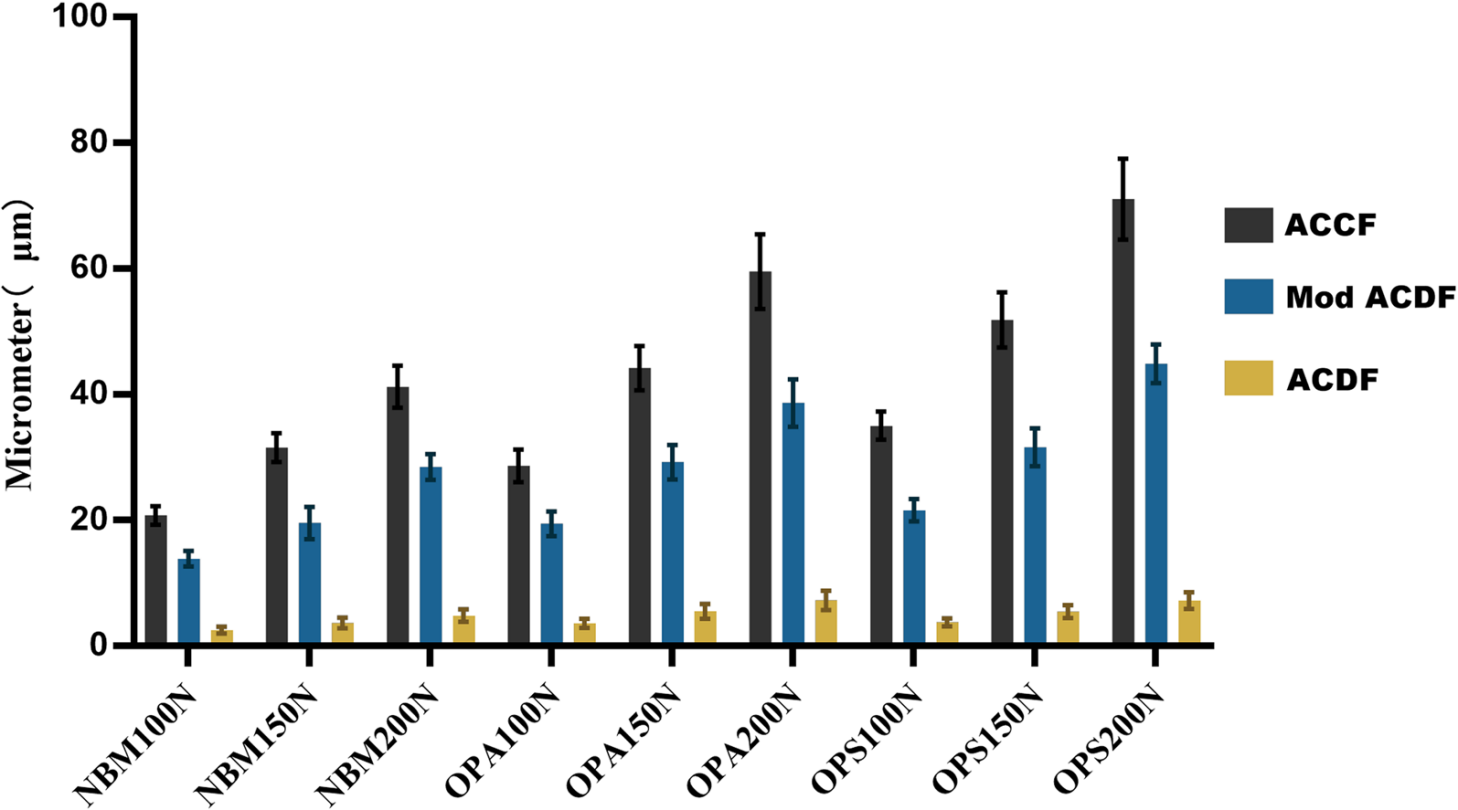
Displacement under different conditions

According to the results of the finite element analysis (Fig. 3a–c), the stresses were mainly distributed along the rigid structure and concentrated on the cortical bone, titanium mesh, titanium plate, and screws (Fig. 4). The magnitude of stress increases with the increase in force, with increases in the standard deviation level and stress distribution deviation. As the bone volume gradually decreases, the stress decreases, the standard deviation level increases, and the deviation intensifies (Table 2). The present study focused on three variables (operative style, bone volume, and force magnitude); thus, we set different variable conditions and compared the remaining two variables for cross-sectional correlation (Figs. 5 and 6).

**Table 2:** Von Mises stress and displacement under different conditions

Surgery	Bone mass	Von Mises stress (MPa)			Displacement (μm)		
		100 N	150 N	200 N	100 N	150 N	200 N
ACCF	NBM	5.80 ± 1.89	7.93 ± 1.81	10.82 ± 2.65	20.41(0.63)	31.14 (1.15)	40.09(2.43)
	OPA	4.76 ± 1.54	8.09 ± 2.37	9.79 ± 2.79	27.86(0.96)	43.70 (2.31)	59.14(6.11)
	OPS	5.70 ± 2.21	8.96 ± 3.43	11.56 ± 4.53	34.32(0.36)	50.35 (0.63)	68.86(1.27)
Mod ACDF	NBM	3.03 ± 1.07	4.21 ± 1.67	6.13 ± 2.26	13.91 ± 1.25	18.23 (4.58)	27.57(3.79)
	OPA	2.80 ± 1.04	4.23 ± 1.86	5.65 ± 2.19	19.47 ± 1.95	29.27 (4.32)	36.89(7.11)
	OPS	3.07 ± 1.12	4.97 ± 1.69	6.68 ± 2.36	21.62 ± 1.76	30.75 (5.50)	44.61(6.21)
ACDF	NBM	0.73 ± 0.25	1.04 ± 0.42	1.41 ± 0.51	2.57 ± 0.52	3.67 (1.76)	4.86 ± 0.98
	OPA	0.67 ± 0.22	1.07 ± 0.43	1.32 ± 0.49	3.67 ± 0.73	5.86 (2.36)	7.31 ± 1.53
	OPS	0.61 ± 0.19	0.99 ± 0.32	1.29 ± 0.40	3.80 ± 0.65	5.77 (2.07)	7.28 ± 1.32

### Comparison of stress and deformation under different forces

In the three ACCF model groups with different bone weights (Table 3), the overall Von Mises Stress magnitude fluctuated between 4.76 and 11.56 MPa. The largest difference appeared when comparison 100 N with 200 N force, which was significantly different in the three groups with different bone volumes. In the NBM and OPA groups, the Von Mises Stress produced by 150 N vs. 200 N forces differed (7.93 vs. 10.82 MPa, 8.09 vs. 9.79 MPa). In the OPA group, there was a difference in the stresses produced by 100 N vs. 150 N forces (4.76 vs. 8.09 MPa).

**Table 3:** **a** Comparison of Von Mises stress (MPa). **b** Comparison of displacement (μm)

Surgery	NBM			OPA			OPS		
	Comparison	Statistical value	*P* value	Comparison	Statistical value	*P* value	Comparison	Statistical value	*P* value
*a*									
ACCF	100 N:150 N	10.954	0.061	100 N:150 N	104.579	0	100 N:150 N	5.556	0.078
	100 N:200 N		0	100 N:200 N		0	100 N:200 N		0.003
	150 N:200 N		0.041	150 N:200 N		0	150 N:200 N		0.155
Mod ACDF	100 N:150 N	6.464	0.189	100 N:150 N	5.206	0.121	100 N:150 N	8.066	0.047
	100 N:200 N		0.002	100 N:200 N		0.004	100 N:200 N		0.001
	150 N:200 N		0.039	150 N:200 N		0.122	150 N:200 N		0.071
ACDF	100 N:150 N	5.461	0.147	100 N:150 N	5.373	0.059	100 N:150 N	9.219	0.026
	100 N:200 N		0.003	100 N:200 N		0.004	100 N:200 N		0
	150 N:200 N		0.087	150 N:200 N		0.223	150 N:200 N		0.073
*b*									
ACCF	100 N:150 N	20.48	0.071	100 N:150 N	104.579	0	100 N:150 N	5.556	0.078
	100 N:200 N		0	100 N:200 N		0	100 N:200 N		0.003
	150 N:200 N		0.071	150 N:200 N		0	150 N:200 N		0.155
Mod ACDF	100 N:150 N	20.48	0.071	100 N:150 N	20.48	0.071	100 N:150 N	20.48	0.071
	100 N:200 N		0	100 N:200 N		0	100 N:200 N		0
	150 N:200 N		0.071	150 N:200 N		0.071	150 N:200 N		0.071
ACDF	100 N:150 N	16.242	0.009	100 N:150 N	15.26	0.071	100 N:150 N	22.587	0.003
	100 N:200 N		0	100 N:200 N		0	100 N:200 N		0
	150 N:200 N		0.01	150 N:200 N		0.312	150 N:200 N		0.003

In the Mod ACDF group with three different bone weights, the Von Mises Stress magnitude fluctuated between 2.80 and 6.68 MPa. The greatest variability was found in the comparison of 100 N vs. 200 N forces in the three groups; the comparison of 150 N vs. 200 N forces showed a significant difference in the NBM group (4.21 vs. 6.13 MPa), and the comparison of 100 vs. 150 N forces showed a difference in the OPS group (4.97 vs. 6.68 MPa).

Among the three ACDF model groups with different bone volumes, the fluctuation range of Von Mises Stress was smaller (0.61–4.41 MPa) and the difference was significant in OPS group (*P* = 0.026). The comparison of 100 vs. 200 N forces was consistently different, whereas the difference between 150 and 200 N forces was not significant.

There was a significant difference in the deformation in all groups (displacement range: 2.57–71.04 μm), except for the comparison of 150 vs. 200 N forces producing deformation in the ACDF group with a reduced bone volume within the ACDF group (5.57 vs. 7.31 μm, *P* = 0.312). Within the ACCF and Mod ACDF groups, only the comparison between 100 and 200 N was always different. Within the ACCF and OPA groups, differences were observed when comparing different forces.

### Comparison of stress and deformations at different bone volumes

When limiting the surface forces and performing the comparison of the differences in bone volume (Table 4), no significant differences in the stress levels were observed among the ACCF, ACDF, and Mod ACDF groups. Regarding vertebral surface displacement, there were significant differences between the ACCF and NBM groups as compared with the ACCF and OPA groups and between the ACCF and NBM groups, as compared with the ACCF and OPS groups under different forces. The NBM and OPS differences always existed, and differences in deformations between the NBM and OPA groups when acting at 150 and 200 N (3.73 vs. 5.57 μm, 4.86 vs. 7.31 μm). Except for the nonsignificant difference between Mod ACDF-NBM and Mod ACDF-OPA comparisons at 100N force (2.57 μm vs. 3.67 μm), Mod ACDF-NBM vs. Mod ACDF-OPA, Mod ACDF-NBM vs. Mod ACDF-OPS all had differences in the comparison of deformation. Moreover, the comparison of the Mod ACDF-OPA group with the Mod ACDF-OPS group was not significantly different in deformation.

**Table 4:** **a** Comparison of Von Mises stress (MPa). **b** Comparison of Displacement (μm)

Surgery	100 N force			150 N force			200 N force		
	Comparison	Statistical value	*P* value	Comparison	Statistical value	*P* value	Comparison	Statistical value	*P* value
*a*									
ACCF	NBM:OPA	0.735	0.286	NBM:OPA	0.359	0.901	NBM:OPA	0.536	0.556
	NBM:OPS		0.922	NBM:OPS		0.439	NBM:OPS		0.670
	OPA:OPS		0.330	OPA:OPS		0.515	OPA:OPS		0.314
Mod ACDF	NBM:OPA	0.147	0.674	NBM:OPA	0.490	0.983	NBM:OPA	0.410	0.681
	NBM:OPS		0.939	NBM:OPS		0.395	NBM:OPS		0.631
	OPA:OPS		0.619	OPA:OPS		0.407	OPA:OPS		0.376
ACDF	NBM:OPA	0.584	0.599	NBM:OPA	0.078	0.885	NBM:OPA	0.135	0.708
	NBM:OPS		0.292	NBM:OPS		0.810	NBM:OPS		0.625
	OPA:OPS		0.590	OPA:OPS		0.701	OPA:OPS		0.908
*b*									
ACCF	NBM:OPA	18.485	0.036	NBM:OPA	18.485	0.036	NBM:OPA	18.485	0.036
	NBM:OPS		0.000	NBM:OPS		0.000	NBM:OPS		0.000
	OPA:OPS		0.231	OPA:OPS		0.231	OPA:OPS		0.231
Mod ACDF	NBM:OPA	17.165	0.019	NBM:OPA	16.485	0.013	NBM:OPA	18.240	0.033
	NBM:OPS		0.000	NBM:OPS		0.000	NBM:OPS		0.000
	OPA:OPS		0.537	OPA:OPS		0.867	OPA:OPS		0.269
ACDF	NBM:OPA	8.435	0.065	NBM:OPA	9.035	0.036	NBM:OPA	10.145	0.019
	NBM:OPS		0.022	NBM:OPS		0.022	NBM:OPS		0.016
	OPA:OPS		1.000	OPA:OPS		1.000	OPA:OPS		1.000

### Comparison of stress and deformation between different techniques

When comparing the differences in stress and deformation between the different techniques (Table 5), significant differences in stress were observed among the three groups, which were limited to different bone volumes and forces. Regarding deformation, a significant difference was observed between ACCF and ACDF at different bone volumes and forces. The degree of deformation in the Mod ACDF group was between ACCF and ACDF, but there was no significant difference.

**Table 5:** **a** Comparison of Von Mises stress (MPa). **b** comparison of displacement (μm)

Bone mass	100 N force			150 N force			200 N force		
BMD	Comparison	Statistical value	*P* value	Comparison	Statistical value	*P* value	Comparison	Statistical value	*P* value
*a*									
NBM	ACCF:Mod	32.223	0	ACCF:Mod	45.635	0	ACCF:Mod	42.838	0
	ACCF:ACDF		0	ACCF:ACDF		0	ACCF:ACDF		0
	ACDF:Mod		0.002	ACDF:Mod		0	ACDF:Mod		0
OPA	ACCF:Mod	28.558	0.002	ACCF:Mod	32.11	0	ACCF:Mod	33.541	0.001
	ACCF:ACDF		0	ACCF:ACDF		0	ACCF:ACDF		0
	ACDF:Mod		0.001	ACDF:Mod		0.002	ACDF:Mod		0
OPS	ACCF:Mod	25.343	0.001	ACCF:Mod	25.858	0.002	ACCF:Mod	24.11	0.003
	ACCF:ACDF		0	ACCF:ACDF		0.000	ACCF:ACDF		0
	ACDF:Mod		0.002	ACDF:Mod		0.002	ACDF:Mod		0.002
*b*									
NBM	ACCF:Mod	20.48	0.071	ACCF:Mod	20.48	0.071	ACCF:Mod	20.48	0.071
	ACCF:ACDF		0	ACCF:ACDF		0	ACCF:ACDF		0
	ACDF:Mod		0.071	ACDF:Mod		0.071	ACDF:Mod		0.071
OPA	ACCF:Mod	20.48	0.071	ACCF:Mod	20.48	0.071	ACCF:Mod	20.48	0.071
	ACCF:ACDF		0	ACCF:ACDF		0	ACCF:ACDF		0
	ACDF:Mod		0.071	ACDF:Mod		0.071	ACDF:Mod		0.071
OPS	ACCF:Mod	20.48	0.071	ACCF:Mod	20.48	0.071	ACCF:Mod	20.48	0.071
	ACCF:ACDF		0	ACCF:ACDF		0	ACCF:ACDF		0
	ACDF:Mod		0.071	ACDF:Mod		0.071	ACDF:Mod		0.071

## Discussion

Posterior longitudinal ligament ossification of the cervical spine is a rare disease with a high prevalence in Asia [16]. According to the Japanese Ministry of Health, Labour, and Welfare [17], the prevalence of OPLL is high in Japan and China, reaching an incidence of 1.1–1.7%. The cause of OPLL is still unclear [18], and most cases of ligamentous ossification are mild and do not cause excessive clinical symptoms. Contrarily, for OPLL causing severe symptoms, the treatment options include direct surgical resection or indirect decompression of the spinal canal. Many surgical treatment options for different types of cervical OPLL have been reported previously [19–21], and considerable advantages and disadvantages were reported among these interventions, such as ACDF resection for focal OPLL near the level of the intervertebral space and ACCF resection for segmental or continuous OPLL. Recently, the use of special surgical techniques for OPLL with more accumulated segments was described, and the recovery of cervical motion postoperatively was found to better than that preoperatively [22–24]. However, for OPLL at the central vertebral level, no cases of direct resection have been reported.

In a previous clinical study [12], we proposed a novel technique that was based on the modification of ACDF, i.e., partial osteotomy of the vertebral body via the anterior approach and direct access to the posterior edge of the vertebral body to remove the ossified compression. In a preconceived scenario, this technique would not be limited by a narrow field of view or cause excessive bone damage and would obtain a superior range of motion in the cervical spine by fixing fewer segments. Therefore, we believe that this new technique should only be used in patients with a good bone density but with poor OPLL distribution location. However, this technique still raises concerns among some orthopedic surgeons: can the patient’s vertebral body tolerate the altered mechanical conduction caused by the Mod ACDF osteotomy? To the best of our knowledge, the differences in bone density are a cause of fusion subsidence, and several literature reviews [25–27] have reported that low bone mass is an important risk factor for fusion subsidiarity. Moreover, this new technique has not yet theoretically validated the relative influence of bone density on vertebral morphological stability. With this question in mind, we designed three model groups with different techniques and bone masses and analyzed and explored the different working conditions of each model based on the system of a real physics engine.

Most of the load applied to the spinal unit is concentrated on the vertebral body, with a small portion of the load dispersed to the articular processes on both sides. According to the three-column theory proposed by Dennis and its derivatives, the vertebral structure of the spine carries the majority of the axial stress. Because of the characteristics of vertebral body movement, the posterior side of the vertebral body is significantly higher than the anterior side. Therefore, the load-bearing capacity of the vertebral body is highly correlated with the spine integrity. The shape of the vertebral body can be described as a cylindrical structure with the cortical bone as a hard shell wrapped around the “soft” cancellous bone. Based on the stress diagrams developed for the skeletal model, when the difference in Young's modulus between the rigid shell and the inner filling is large enough, the filling cancellous bone deforms in parallel with the outer cortical bone, but the magnitude of the stress is not significant. This implies that the load on the vertebral body is transmitted downward by diffusion through a rigid structure composed of cortical bone.

Therefore, on the conventional ACDF model established in this study, when the cage’s contact surface has fully adhered to the bilateral bone and a piece of rigid material is set on the anterior side of the vertebral body to share the anterior load of the vertebral body, the static vertical load will be more concentrated on the mid-posterior part of the upper surface of the vertebral body (Fig. 2a) and transmitted downward along the rigid cortical bone shell. In the data description of stresses, the standard deviation of stresses within the ACDF group floated from 0.19 to 0.51, with the smallest data difference, and the stresses were more uniformly distributed under different loads and bone volumes and were significantly smaller than those of the other two groups. When the cancellous and cortical bones in the model group were set to a smaller bone volume, the magnitude of stresses decreased and was accompanied by a load sharing of the anterolaterally implanted rigid structure. While observing the deformation of the upper surface of the vertebral body under different conditions, the deformation ranged from 2.57 to 7.28 μm, which was roughly proportional to the bone volume and magnitude of the load. However, the deformation degree was slightly higher in the OPA group with the application of 150 and 200 N than in the OPS group, and we considered that this might be related to the additional weight-bearing of the titanium plate and screws.

In the ACCF operative model, most of the load above was concentrated on the titanium mesh (Fig. 4b) and radiated downward along the titanium mesh toward the eight contact points on the upper surface of C6. Contrarily, due to the rigid structure of the screw and titanium plate, the stresses on the anterior side of the vertebral body do not show considerably and are mainly distributed on the mid-posterior side. The connection between stress non-concentration and fusion device subsidence has been previously reported [28]. Although titanium mesh-bone surface angulation has a positive effect on stress concentration, to exclude any relevant interference in the present study, we used a titanium mesh model that completely fits the bone surface. Our results show that the mechanical distribution at points 1, 2, 3, and 4 has a greater weight among the eight stress-bearing points. This difference reached its maximum in the OPS group with 200 N applied (standard deviation: 1.54–4.53), which was significantly higher than that of the ACDF group. The degree of deformation at the vertebral contact points was higher in the ACCF group than in the ACDF group (*P* < 0.05), regardless of the bone volume performance, according to the ANSYS deformation degree report, a finding consistent with previous studies, which we consider to have originated from the fusion disparity in the contact area.

In the Mod ACDF technique description, when we remove the rectangular slot directly, part of the structure that can share the overlying stresses disappears, and this stress will be compensated by the remaining bone. In the original intact vertebral body, the stresses can be evenly distributed on the lower surface by a rigid vertebral shell with a uniform texture. In the new partially osteotomized model of the vertebral body, however, the stresses are mostly concentrated on the four posterior vertices of the rectangular notch and transverse edges due to the presence of the anterior titanium plate of the vertebral body. The loss of a part of the rigid shell is fixed to the anterior rigid structure, which leads to an uneven conduction on the surface of the diseased vertebra and enriches the stresses on the posterior side. According to the structural characteristics of a rectangular osteotomy profile, the sharp rectangular outer corners will become the concentration of stresses, which will increase the probability of physical material deformation. The results showed that the stresses in the normal bone size osteotomized the vertebrae under 100 N load fluctuated from 1.764 to 4.728 MPa, with the peak stress occurring at point 2. The magnitude of the stresses increased as the magnitude of the applied load increased. While the results of comparing the stress magnitudes of the three groups under the same size of the applied force conditions were not intuitive, the stresses in the OPS group were greater than those of the other two groups, but the differences were not significant. According to the material deformation report, the deformation of Mod ACDF was concentrated in the circular region covered by a cage and the maximum deformation occurred at the center of the circle (31.932–49.053 μm). The degree of deformation in the NBM group was significantly lower than that of the OPA and OPS group under different magnitudes of forces (*P* < 0.05). Moreover, in the comparison between groups, the degree of deformation in the Mod ACDF group was higher than that of the ACDF group and lower than that of the ACCF group, but the difference was not significant (*P* = 0.071).

The results suggest that the risk of fusion device subsidence in the Mod ACDF group is between ACDF and ACCF. Bone loss not only affects the stress distribution of the unit vertebrae but also increases the risk of vertebral collapse. The probability of such adverse events will increase especially after a strong intervention of vertebral morphology by using relevant techniques, such as ACCF and Mod ACDF.

Therefore, Mod ACDF combined with anterior fixation of the plate is the best surgical option for the treatment of OPLL in a specific position. The comparison with other anterior cervical techniques can be summarized into the following two points: Compared to ACDF, the stresses are enriched at the posterior vertex and transverse border due to the additional resection of the cortical bone, with higher peak stresses than ACDF (49.053 vs. 9.302 μm) and higher peak deformation than ACDF (49.053 vs. 9.302 μm), with a higher probability of vertebral collapse as compared to ACDF. Compared with ACCF, the peak stress and deformation levels were lower (10.764 vs. 18.561 MPa; 49.053 vs. 86.863 μm), minimizing bone damage to the body, with fewer fixed segments, better postoperative segmental Cobb angle, and better patient satisfaction feedback.

## Limitations

Finite element analysis occupies an important place in spine surgery, which has high biomechanical requirements, by simulating the infinite unknown quantities of reality through finite units based on real physical systems. However, as far as the use of FEA in this study is concerned, it still has the following shortcomings: individual variability cannot be ignored, cervical anatomy and bony variability can still occur, and differences between models may lead to deviations in the final results; the differences in cervical curvature may affect the mechanical distribution of loads understanding conditions due to the prone position of the volunteers during the filming; due to constraints imposed by health insurance and bed availability, it was not feasible to conduct a preoperative finite element simulation evaluation for this surgical technique type. However, based on previous case reports, we have determined that this patient could potentially achieve a favorable outcome, supported by imaging and clinical evidence. The findings presented in this study provide supplementary evidence regarding the theoretical feasibility of Mod ACDF, and they do not influence the final outcome; this study only focuses on the natural effects of vertebral body collapse due to gravity, and the stress, and deformation effects of different loads on the vertebral body under motion conditions, such as forward flexion, back extension, left rotation, and right rotation are still lacking. In the future, we will fully refer to the real motion system and do a systematic review of the stresses on bilateral articular processes and vertebral body surfaces; and we only explored the mechanical characteristics of different techniques under different bone volumes and forces, and did not set destructive load values; the results are only applicable to experiments, and the effects of other conditions should be fully considered when applying the results to clinical practice.

## Conclusions

Under the influence of different loads and bone volumes, the stress distribution of the vertebral body after Mod ACDF was worse than that after ACDF and better than that of ACCF. The degree of deformation of the vertebral body surface was between ACCF and ACDF. Our study data suggest that the Mod ACDF technique has less impact on cervical spine biomechanics as compared to ACCF and it has led to lesser bone destruction and higher patient satisfaction, which is particularly suitable for cervical OPLL cases in special locations.

## Data Availability

The datasets used and analyzed during the current study are available from the corresponding author on reasonable request.

## Abbreviations

ACDF: Anterior cervical discectomy and fusion
ACCF: Anterior cervical corpectomy and fusion
ModACDF: Modified anterior cervical discectomy and fusion
NBM: Normal bone mineral density
OPA: Osteopenia
OPS: Osteoporosis
OPLL: Ossification of the posterior longitudinal ligament
CT: Computed tomography
FEA: Finite element model
