# Correction: Static mechanical analysis of the vertebral body after modified anterior cervical discectomy and fusion (partial vertebral osteotomy): a finite element model

**DOI:** 10.1186/s13018-023-04107-7

**Published:** 2023-09-09

**Authors:** Huo‑huo Xue, Dian Tang, Wen‑han Zhao, Liang Chen, Zhong Liao, Jing‑lai Xue

**Affiliations:** 1 Fujian Medical University Union Hospital, Fuzhou, 350100 China; 2 Department of Spine Surgery, Fuzhou Second Hospital, Fuzhou, 350007 China

**Correction: Journal of Orthopaedic Surgery and Research (2023) 18:554** **https://doi.org/10.1186/s13018-023-04033-8**

Following publication of the original article [1], the affiliations of authors “Huo‑huo Xue, Dian Tang, Zhong Liao, Jing-lai Xue” have been changed to “Department of spine surgery, Fuzhou Second Hospital, 350007 Fuzhou, China”

The author would like to update the funding information as “Fujian Provincial Clinical Medical Research Center for First Aid and Rehabilitation in Orthopaedic Trauma (2020Y2014)”.

## References

[1] Xue H (2023). Static mechanical analysis of the vertebral body after modified anterior cervical discectomy and fusion (partial vertebral osteotomy): a finite element model. J Orthop Surg Res.

